# Supplementary material for: Reconciling Work and Family Demands and Related Psychosocial Risk and Support Factors among Working Families: A Finnish National Survey Study
Source: Int J Environ Res Public Health. 2022 Jul 13;19(14):8566. doi: 10.3390/ijerph19148566 (PMC9318108; doi:10.3390/ijerph19148566)
Supplement: Supplementary file 1 [file ijerph-19-08566-s001.zip › ijerph-1794802-supplementary.pdf]

International Journal of
Environmental Research and Public Health

MDPI

Supplementary Materials

Table S1. The recoding process.

Original Variable	Response Options	Recoding
Work-family conflict		
Family interference with work (FIW):	'Totally true', 'True to some extent', 'Untrue to some extent'	Reported FIW
'Matters at home at times disturb concentration on my work'	'Totally untrue'	Reported no FIW
Work interference with family (WIF):	'Totally true', 'True to some extent', 'Untrue to some extent'	Reported WIF
'I feel that I am neglecting home matters because of my job'	'Totally untrue'	Reported no WIF
Socio-demographic and workplace characteristics		
Chronological age	15–19 [a]	
	20–24, 25–29, 30–34	20–34
	35–39, 40–44	35–44
	45–49, 50–54	45–54
	55–59, 60–64, 65–67	55–67
Gender	Man	*
	Woman	
Educational level	Tertiary level or university education	High
	Polytechnic education, Vocational college education, Specialist vocational qualification, General upper secondary school, Vocational school education, further vocational qualification, or vocational upper secondary qualification, Lower secondary or comprehensive school education, Primary education or less	Low
Age of children	Chronological age (years)	0–7 years only
		8–17 years only
		Mixed
Temporal work flexibility: 'Do you have strictly set starting and finishing times for your work, or can you personally influence them by at least 30 minutes either way?'	Flexible: 'Able to influence starting and finishing times (e.g. flexitime)'	*
	Fixed: 'Set starting and finishing times'	
Spatial work flexibility: 'Do you do telework?'	'I do at present'	Telework
	'No, but I have done before'	No telework
	'No, I do not'	
Employment type	Part-time	*
	Full-time	
Number of subordinates	Exact number of subordinates	No subordinates
		1–9
		10 or more
Psychosocial work factors		
Overtime: 'I often have to stretch my working day to get all the work done'	'Totally true', 'True to some extent'	Agree
	'Untrue to some extent', 'Totally untrue'	Disagree
Task overload: 'I usually have too many different tasks under way'	'Totally true', 'True to some extent'	Agree
	'Untrue to some extent', 'Totally untrue'	Disagree
	'Totally true', 'True to some extent'	Agree

Work pace: 'I do not have time to do my work as well and conscientiously as I would like to'	'Untrue to some extent', 'Totally untrue'	Disagree
Superior support: 'When your work seems difficult, do you receive support and encouragement from your superiors?'	'Never'<br>'Sometimes'<br>'Often'<br>'Always'	*
Co-worker support: 'When work seems difficult, do you receive support and encouragement from your co-workers?'	'Never'<br>'Sometimes'<br>'Often'<br>'Always'	*
Psychosocial family factors		
Only part-time work: 'Have you sometimes during your professional career worked only part-time for family reasons?' [b]	'No'<br>'Yes'	*
Task reduction: 'Have you sometimes during your professional career cut down on your job tasks for family reasons?' [b]	'No'<br>'Yes'	*
Refused more work demands: 'Have you sometimes during your professional career refused more demanding jobs or tasks for family reasons?' [b]	'No'<br>'Yes'	*
Family support (when demands are conflicting): 'Has there been conflicts in your family about working hours, household work and personal time?'	Frequent conflicts: 'There are a lot of conflicts and battles about time'<br>Occasional conflicts: 'Conflicts arise from time to time'<br>No conflicts anymore: 'There were conflicts before but not anymore'<br>No conflicts: 'No, the times are adjusted peaceably'	*
Support from close ones: 'Combining work and family is made easier for me with the help I get from family or friends'	'Totally true', 'True to some extent'	Agree
	'Untrue to some extent', 'Totally untrue'	Disagree

'Not applicable' was a possible response option to all items (except for the control variables and the item measuring family support), and 'Cannot say' to all items measuring work factors. [a] No respondent adhered to this age group in the current study sample. [b] 'Family' was used in a wide meaning and included other close relatives to the respondent. * No recoding: the original variables were analyzed.

Table S2. Overview of the study sample according to variables measuring psychosocial work and family factors. $N = 1431$.

Variable	Response Category	*N* (%)
Psychosocial work factors		
Overtime	Agree	580 (40.5)
	Disagree	827 (57.8)
	N/A	24 (1.7)
	Cannot say	0 (0)
Task overload	Agree	753 (52.6)
	Disagree	665 (46.5)
	N/A	13 (0.9)
	Cannot say	0 (0)

Work pace	Agree	611 (42.7)
	Disagree	812 (56.7)
	N/A	8 (0.6)
	Cannot say	0 (0)
Superior support	Never	90 (6.3)
	Sometimes	410 (28.7)
	Often	558 (39.0)
	Always	355 (24.8)
	N/A	17 (1.2)
	Cannot say	1 (0.1)
Co-worker support	Never	25 (1.7)
	Sometimes	230 (16.1)
	Often	612 (42.8)
	Always	551 (38.5)
	N/A	13 (0.9)
	Cannot say	0 (0)
Psychosocial family factors		
Only part-time work	Yes	409 (28.6)
	No	1014 (70.9)
	N/A	7 (0.5)
Task reduction	Yes	475 (33.2)
	No	953 (66.6)
	N/A	2 (0.1)
Refused more work demands	Yes	319 (22.3)
	No	1107 (77.4)
	N/A	4 (0.3)
Family support	Frequent conflicts	64 (4.5)
	Occasional conflicts	792 (55.3)
	No conflicts anymore	13 (0.9)
	No conflicts	550 (38.4)
Support from close ones	Disagree	593 (41.4)
	Agree	754 (52.7)
	N/A	80 (5.6)
	Cannot say	3 (0.2)

Missing data ranged from 0 (0%) to 3 (0.002%) for the included variables. N/A = Not applicable. After initial, descriptive analyses, 'not applicable-' and 'cannot say-' responses were excluded.

Table S3. The distribution and between-group comparison of psychosocial work and family factors among participants according to reported family interference with work (FIW)/work interference with family (WIF) status. $N = 1\,431$.

	FIW (%)	No FIW (%)	$\chi 2$	WIF (%)	No WIF (%)	$\chi 2$
Psychosocial work factors						
Overtime			$p \leq 0.001$			$p \leq 0.001$
Agree	427 (74.5)	146 (25.5)		497 (86)	81 (14)	
Disagree	522 (63.6)	299 (36.4)		535 (65.1)	287 (34.9)	
Task overload			$p \leq 0.001$			$p \leq 0.001$
Agree	560 (74.9)	188 (25.1)		629 (84)	120 (16)	
Disagree	396 (60.5)	259 (39.5)		408 (61.7)	253 (38.3)	
Work pace			$p \leq 0.001$			$p \leq 0.001$
Agree	451 (74.4)	155 (25.6)		508 (83.4)	101 (16.6)	
Disagree	508 (63.4)	293 (36.6)		534 (66.2)	273 (33.8)	

Superior support			$p \leq 0.001$			$p = 0.015$
Never	53 (58.9)	37 (41.1)		60 (67.4)	29 (32.6)	
Sometimes	283 (69.5)	124 (30.5)		306 (74.8)	103 (25.2)	
Often	415 (74.9)	139 (25.1)		427 (76.9)	128 (23.1)	
Always	201 (58.1)	145 (41.9)		240 (68.2)	112 (31.8)	
Co-worker support			$p = 0.255$			$p = 0.273$
Never	15 (60)	10 (40)		18 (72)	7 (28)	
Sometimes	153 (67.1)	75 (32.9)		171 (75)	57 (25)	
Often	429 (70.8)	177 (29.2)		460 (75.9)	146 (24.1)	
Always	358 (65.9)	185 (34.1)		391 (71)	160 (29)	
Psychosocial family factors						
Only part-time work			$p \leq 0.001$			$p = 0.398$
Yes	304 (75.2)	100 (24.8)		305 (74.9)	102 (25.1)	
No	654 (65.1)	350 (34.9)		734 (72.7)	275 (27.3)	
Task reduction			$p \leq 0.001$			$p \leq 0.001$
Yes	350 (74.5)	120 (25.5)		378 (79.9)	95 (20.1)	
No	611 (64.8)	332 (35.2)		666 (70.3)	282 (29.7)	
Refused more work demands			$p = 0.007$			$p \leq 0.001$
Yes	235 (74.4)	81 (25.6)		259 (81.7)	58 (18.3)	
No	726 (66.3)	369 (33.7)		783 (71.1)	319 (28.9)	
Family support			$p \leq 0.001$			$p \leq 0.001$
Frequent conflicts	58 (90.6)	6 (9.4)		53 (82.8)	11 (17.2)	
Occasional conflicts	602 (76.6)	184 (23.4)		641 (81.3)	147 (18.7)	
No conflicts anymore	8 (66.7)	4 (33.3)		11 (91.7)	1 (8.3)	
No conflicts	287 (53)	254 (47)		332 (60.6)	216 (39.4)	
Support from close ones			$p = 0.860$			$p = 0.264$
Disagree	406 (69.2)	181 (30.8)		427 (72.1)	165 (27.9)	
Agree	514 (68.7)	234 (31.3)		562 (74.8)	189 (25.2)	
